# Data Monitoring for the Chinese Clinical Trials of Remdesivir in Treating Patients with COVID-19 During the Pandemic Crisis

**DOI:** 10.1007/s43441-020-00159-7

**Published:** 2020-05-16

**Authors:** Weichung J. Shih, Chen Yao, Tai Xie

**Affiliations:** 1 Rutgers University School of Public Health, Piscataway, NJ USA; 2 Peking University Clinical Research Institute, Beijing, China; 3 Brightech International and CIMS Global LLC, Somerset, NJ USA

**Keywords:** Monitoring clinical trials, ICH, DSMB, eDMC, DDM, Remdesivir, COVID-19 pandemic

## Abstract

Two phase-III, double-blind, randomized clinical trials of remdesivir plus SOC (standard of care) versus placebo plus SOC have been conducted in Wuhan hospitals by Chinese investigators during the urgent COVID-19 epidemic [ClincalTrials.gov NCT04257656 and NCT04252664]. These trials have been highly anticipated worldwide. We expect investigators of the trials will soon report the clinical and laboratory findings from the medical perspective. This manuscript provides documentary style information on the process of monitoring key data and making recommendations to the sponsor and investigators based on analytical insights when dealing with the emergent situation from the statistical viewpoint. Having monitored data sequentially from 237 patients, we comment on the strength and weakness of the study design and suggest the treatment effect of remdesivir on severe COVID-19 cases. Our experience with using the Dynamic Data Monitoring (DDM) tool has demonstrated its efficiency and reliability in supporting DSMB’s instantaneous review of essential data during the emergent situation. DDM, when used properly by disciplined statisticians, has shown its capability of exploring the trial data flexibly and, in the meantime, protecting the trial’s scientific integrity.

## Introduction

During February 3–6, 2020, just a few weeks after the outbreak of the novel coronavirus (nCOV-2019) in the city of Wuhan, Chinese investigators urgently initiated two clinical trials to evaluate the efficacy and safety of remdesivir for treating hospitalized adult patients with respiratory disease caused by the virus, lately known as COVID-19 (WHO formally named it on February 11, 2020). The first trial was to treat patients with severe symptoms [1] and the second one was to treat patients with mild–moderate symptoms [2]. Both trials were randomized, double-blind, placebo-controlled, multicenter studies, sponsored by the Institute of Materia Medica, Chinese Academy of Medical Sciences. To ensure the interests of patients and the objectivity of the trial sponsor and investigators, who were masked with respect to the treatment assignments on patients throughout the study, a Data and Safety Monitoring Board (DSMB) was composed with five members: 2 medical experts, 1 epidemiologist, and 2 statistical experts, all were independent from the trial sponsor and investigators (see acknowledgement for DSMB member names). With the support of a CRO (Clinical Research Organization), contracted by the sponsor for the task of data management, a kick-off DSMB meeting was launched on February 12, 2020, where the study protocols were reviewed and DSMB Charter was approved, in which the subsequent meeting schedules, data format and monitoring guidance were established. For the rest of this paper, the Background section first addresses the feasibility issue and challenges for the DSMB, which were debated and presented to us prior to the formal kick-off meeting. Controversial exchange of opinions between investigators’ consultants and DSMB on selection of endpoints to monitor is also discussed. The Methods section describes the statistical methods, type-I error rate preservation strategy, and the eDMC tool we used for interim data monitoring during this emergent epidemic situation. In the Results section, we use a diary style to document each DMC meeting by calendar date, highlighting the key findings, discussions, and recommendations to the sponsor and investigator team based on data and seemly trends at hand. Finally, in the Conclusion and Discussion section, with our experience in monitoring the key data from 237 patients sequentially, we comment on some weakness and strength of the trial design and suggest the treatment effect of remdesivir on severe COVID-19 cases. The data monitoring experience we gained by serving on this DSMB for the remdesivir trial in China should provide meaningful information for other researchers who are currently conducting COVID-19 trials globally.

## Background

### Feasibility and Necessity Issues and Challenges for the DSMB

The sponsor and investigator team debated whether it is necessary to compose a DSMB for these two globally anticipated trials. Some conjectured that given the epidemic in Wuhan City, enrollment of patients would be so fast that there would be no time or need for interim data monitoring and reviews. However, considering the 10-day treatment period and [up to] 28-day follow-up period and that remdesivir is an investigational drug, DSMB review was deemed feasible and necessary according to the ICH Good Clinical Practice (GCP) guideline [3] and the Chinese regulation on new drug research and development [4]. The DSMB was then commissioned. Given the urgent situation, the sponsor emphasized that the accumulative efficacy and safety information on this trial should be accessible by the DSMB as soon as possible so that the DSMB could make quick and scientifically sound decisions. The real challenge for the DSMB was to be highly efficient in data transmission and to monitor key efficacy and safety data in a very timely and scientific manner. The DSMB chose to use an eDMC system with a statistical tool called DDM (Dynamic Data Monitoring) software; see more description and use of DDM in the sequel.

### Kick-off meeting: February 11, 2020

At the first DSMB (kick-off) meeting, only open session was held on introduction of key personnel, review of synopses of the study protocols, and discussion and approval of the DMC Charter including data monitoring plan. Besides DMC members, CRO data manager, and independent statisticians, other participants included the sponsor (Academy of Medical Sciences officials), principal investigators (clinicians) with their academic consultants, and a representative of Gilead Science Inc. (the drug supplier). The study status report outlined that 136 severely ill patients (out of 453 planned) had already been randomized (2:1 for remdesivir: placebo) in 3 hospitals/centers in Wuhan (out of 10 planned) for protocol [1]. Some centers were ready to enroll mild–moderate cases within two days for protocol [2]. The DSMB hence requested to have the next meeting to review baseline, efficacy, and safety data of the first trial of severe cases within 7–10 days in anticipating its fast enrollment; subsequent data review could be weekly in principle or shorter depending on the patient enrollment.

The key discussion was on what data to monitor and how the recommendation would be based on at the subsequent data review meetings. DSMB requested that baseline characteristics including demographics, SPO2 (serum oxygen) level, and time from onset to randomization (receiving medication)—these were the two major characteristics differentiating severe from mild/moderate COVID-19 cases, study drug exposure, and patient disposition data should be reviewed. Safety data included clinical adverse events (AE) with special attention on serious or high-grade (≥ 3 in severity) AEs. These data were to be displayed without treatment group identification in the open session and with treatment group identification in the closed session where only DSMB members and independent statisticians were present.

For the efficacy data, there was dispute on the endpoints to be monitored and the frequency of monitoring. The protocol design referenced the World Health Organization’s WHO R&D Blueprint [5] and specified the primary efficacy endpoint, which is time to clinical improvement (“TTCI”) censored at Day 28, defined as the time (in days) from randomization of study treatment until a decline of two categories on a six-category ordinal scale of clinical status (1 = discharged; 6 = death). See the complete description of the ordinal scale in the sequel and in the immediate Methods section. The protocol also specified that one interim analysis for efficacy and futility was to be conducted once half of the total number of TTCI events required had been observed. However, the DSMB had concerns on the TTCI. First, for severe cases, whose risk of death would conceivably be high, TTCI would be infinite/undefined for the dead. The censoring rule on 28 days makes no clinical sense for the dead, and would be indifferent between the dead and the lived who still did not reach 2-point improvement by Day 28. Second, patients might decline then rise on the scale along the 28 days; the choice of the first day reaching 2-point decline might not be meaningful if the patient worsened later. Third, patients with baseline score of 2 (hospitalization, not requiring supplemental oxygen) could not possibly improve two categories, but they were not excluded by the protocol. (Note: protocol later revised TTCI to “2-point decline or discharge” after learning our concern). Thus, instead, the DSMB proposed to use a secondary efficacy endpoint specified in the protocol to monitor the study, which was the 6-category ordinal scale itself, stratified by the baseline category, on Days 7, 14, 21, and 28, as the trial progresses.

After hearing the above from the DSMB, the sponsor also expressed dissatisfaction on the one-time interim analysis with TTCI when half of the total number of required events had been observed and commented that this strategy would not really respond to the urgency of the epidemic situation. Hence, the DSMB’s proposal on the dynamic monitoring procedure using the stratified 6-category scale prevailed. More details on this proposed procedure are given in the following Methods section.

## Methods

### The Dynamic Monitoring Procedure for Subsequent DSMB Reviews

While the protocol-specified primary endpoint, TTCI, and the formal one-time interim analysis plan based on TTCI were left intact, the DSMB designed the following monitoring procedure prior to the second meeting held on February 22, 2020.

The endpoint to be monitored was the 6-category ordinal scale stratified by the baseline scale. The ordinal scale specified in the study protocol was: 6 = death; 5 = hospitalization, requiring ECMO and/or IMV; 4 = hospitalization, requiring NIV and/or HFNC therapy; 3 = hospitalization, requiring supplemental oxygen (but not NIV/HFNC); 2 = hospitalization, not requiring supplemental oxygen; 1 = hospital discharge or meets discharge criteria (discharge criteria are defined as clinical recovery, i.e., fever, respiratory rate, oxygen saturation return to normal, and cough relief). At the open session of each DMC meeting, only the overall baseline distribution of the 6-category scale was shown.

The plan for the closed session was to compare the treatment groups with respect to their distributions of the ordinal scale, using the stratified Wilcoxon–Mann–Whitney (WMW) rank-sum test with ties. As the trial progressed, the trend of the test was to be monitored as patients accumulate and treatment days expand. The plan was also to display the distribution data by bar charts and the WMW rank-sum test on a “radar” screen. The “radar” screen is constructed by boundaries of conditional probabilities to show whether the rank-sum test is in “favorable,” “promising,” or “unfavorable” regions. The calculations of the WMW tests and conditional probabilities are facilitated by the Dynamic Data Monitoring (DDM) software in the eDMC system [6]. More details on the regions with associated conditional probabilities are given in the Result section where the DDM “radar” screen is described with graphical displays. The trace of the rank-sum test signals the trend of the trial result from time to time as patients being enrolled. During the early stage of the trial, we expected more data in earlier days and fewer data in later days of follow-up; data examination would be exploratory. Only if a consistent strong signal is indicated by the rank-sum test (i.e., falling in the favorable region), would the formal analysis on the protocol-designed primary endpoint, TTCI, be triggered. As time progressed, we would expect more patients to have longer follow-up data. Most of times, we planned to do exploratory analysis by examining the “radar” graphs. However, in case, it was needed to protect against an inflated false positive rate, especially at the later stage of the trial when sufficient number of patients were enrolled/followed up and we would examine multiple rank-sum tests on Days 7, 14, 21, and 28, the DSMB chose to use Hochberg’s step-wise procedure [7] for protecting an overall alpha at 0.025 (1-sided, or 0.05 2-sided) level for this secondary endpoint.

Since we had no idea when and how many times the TTCI analysis would be triggered, the group sequential flexible alpha-spending function approach [8] was designed to maintain the overall alpha of 0.025 (1-sided, or 0.05 2-sided) level for the primary endpoint as well. Moreover, anticipating the fast-pace enrollment and relatively short trial duration, and considering the urgent matter for the study, the DSMB chose the Pocock-type alpha-spending function for this primary endpoint. Note that the Pocock-type alpha-spending function being concave rather than convex, indicating that more alpha would be spent at earlier than later time, fits the urgent situation of the epidemic; see a previous discussion on choosing alpha-spending function in [9].

## Results

### The Second DSMB Meeting (First Data Review): February 22, 2020

There were 231 patients consented and 215 randomized (144 in the remdesvir group and 71 in the placebo group). Fifty-four (25.1%) completed the 10 days treatment and none reached 14-day follow-up time yet. Ninety-six (44.7%) were within the 10-day treatment period; 53 (24.7%) did not start treatment yet. Twelve (5.6%) discontinued the treatment due to reasons including AE (*n* = 8) and other (*n* = 4).

**Figure 1:**
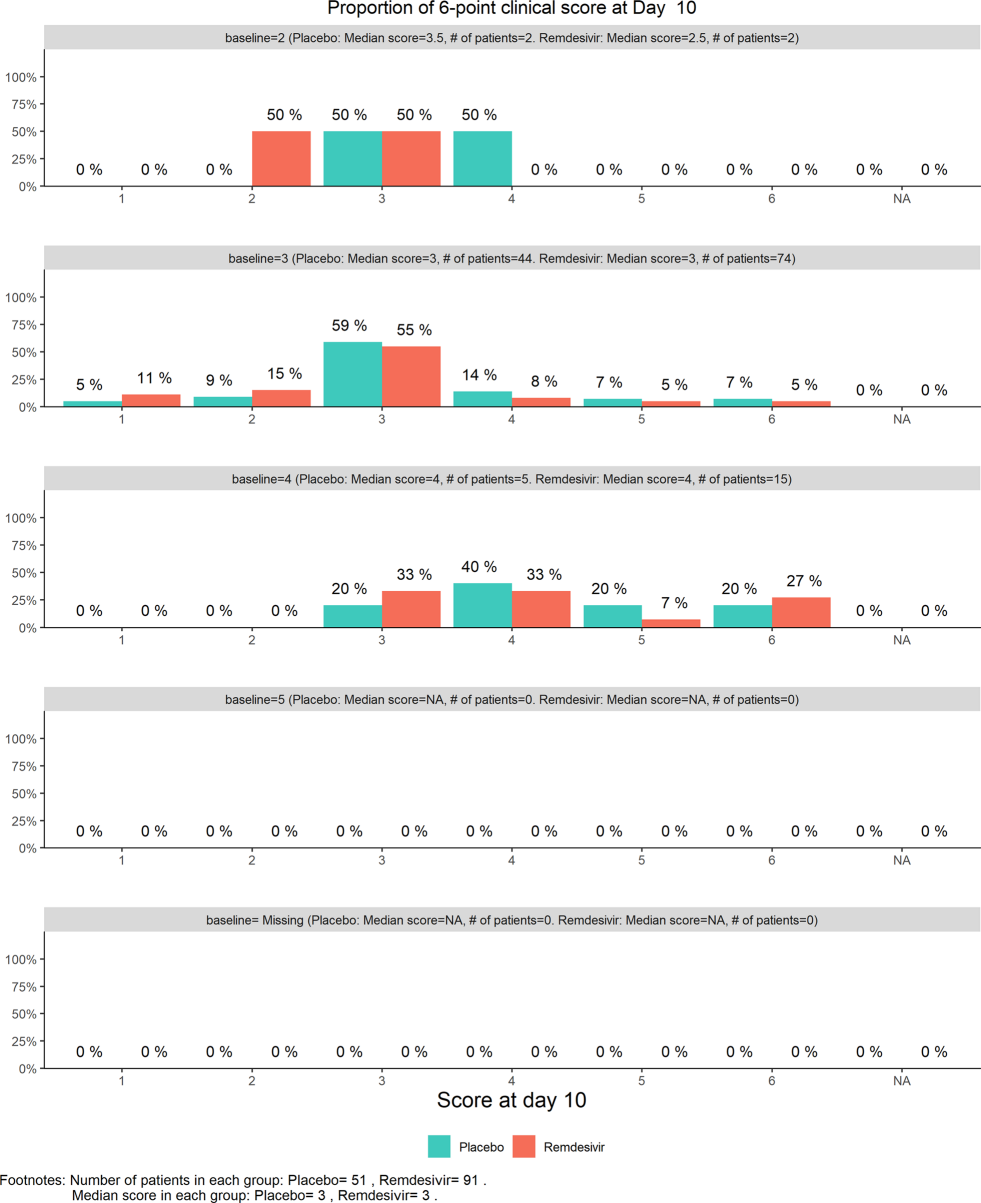
DSMB First Data Review: Distribution of 6-Category Scale Score by Baseline Score at Day 10. Green Color is Placebo, Red Color is Remdesivir. Number of Patients in Each Group: Placebo 51, Remdesivir 91. Median Score in Each Group: Placebo 3, Remdesivir 3.

In the closed session where unblinded data were examined by the DSMB, baseline characteristics and AE proportions were similar between the two groups. No unexpected adverse events were observed, judged by the medical experts of the DSMB. The stratified clinical 6-category scale results were examined with the bar charts and with the stratified WMW rank-sum test displayed on the dynamic “radar” screen. For example, Fig. 1 shows the bar chart for Day 10 results. (Additional bar charts were also requested by the DSMB for Days 3, 5, and 7, but not shown here.) Most (*n* = 118, or 83%) patients’ baseline ordinal category = 3. Four patients’ baseline category = 2, 20 patients’ baseline category = 4, and none were category = 5.

**Figure 2:**
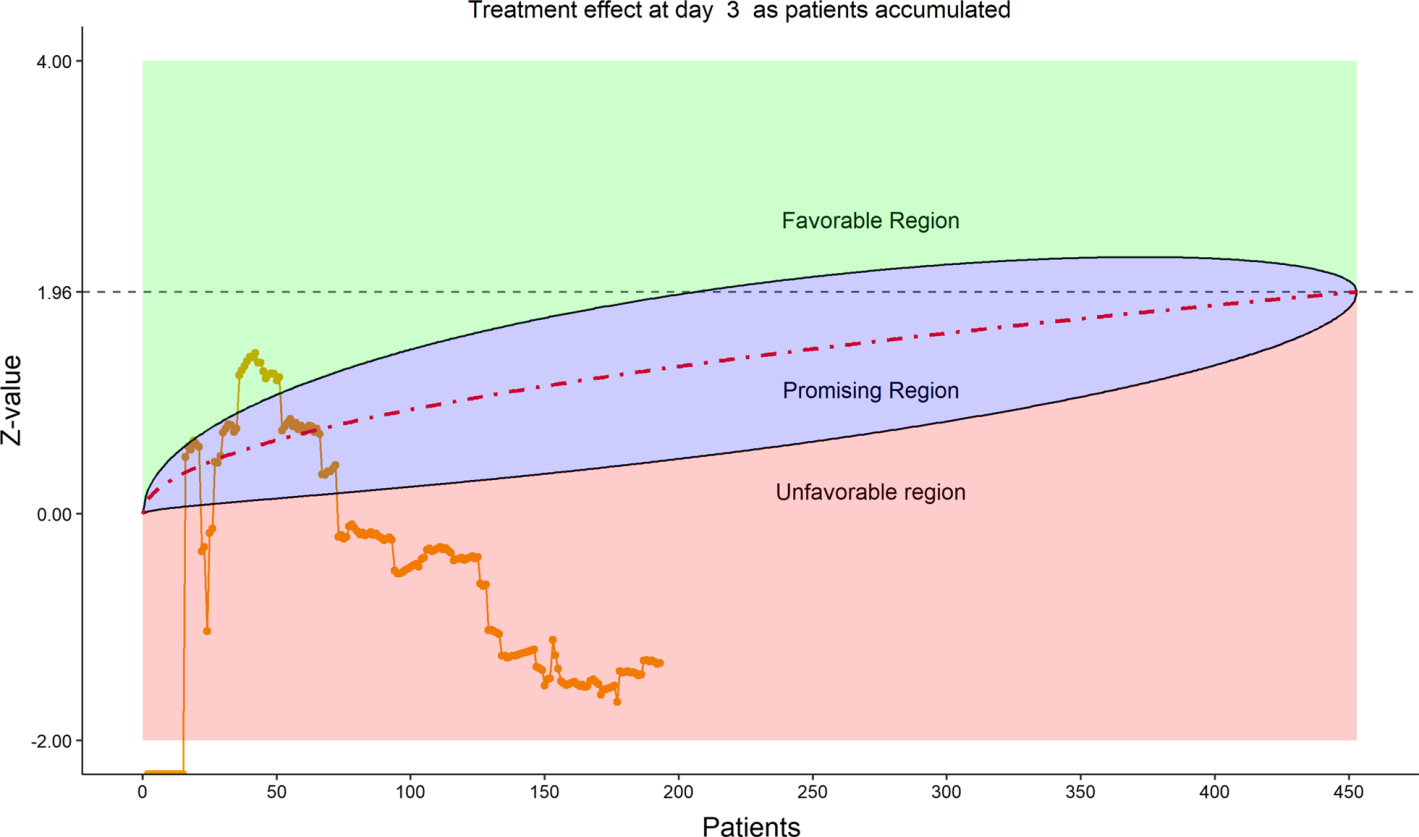
DSMB First Data Review: Dynamic Data Monitoring Z-Score from Stratified Wilcoxon–Mann–Whitney Rank-Sum Test Along Number of Patients Enrolled, on Day 3; The Upper Boundary of the “Promising Zone” Was Set for Conditional Probability (CP) = 90% and the Lower Boundary Was Set for CP = 5%. The Middle Dash Line Represented CP = 50%

**Figure 3:**
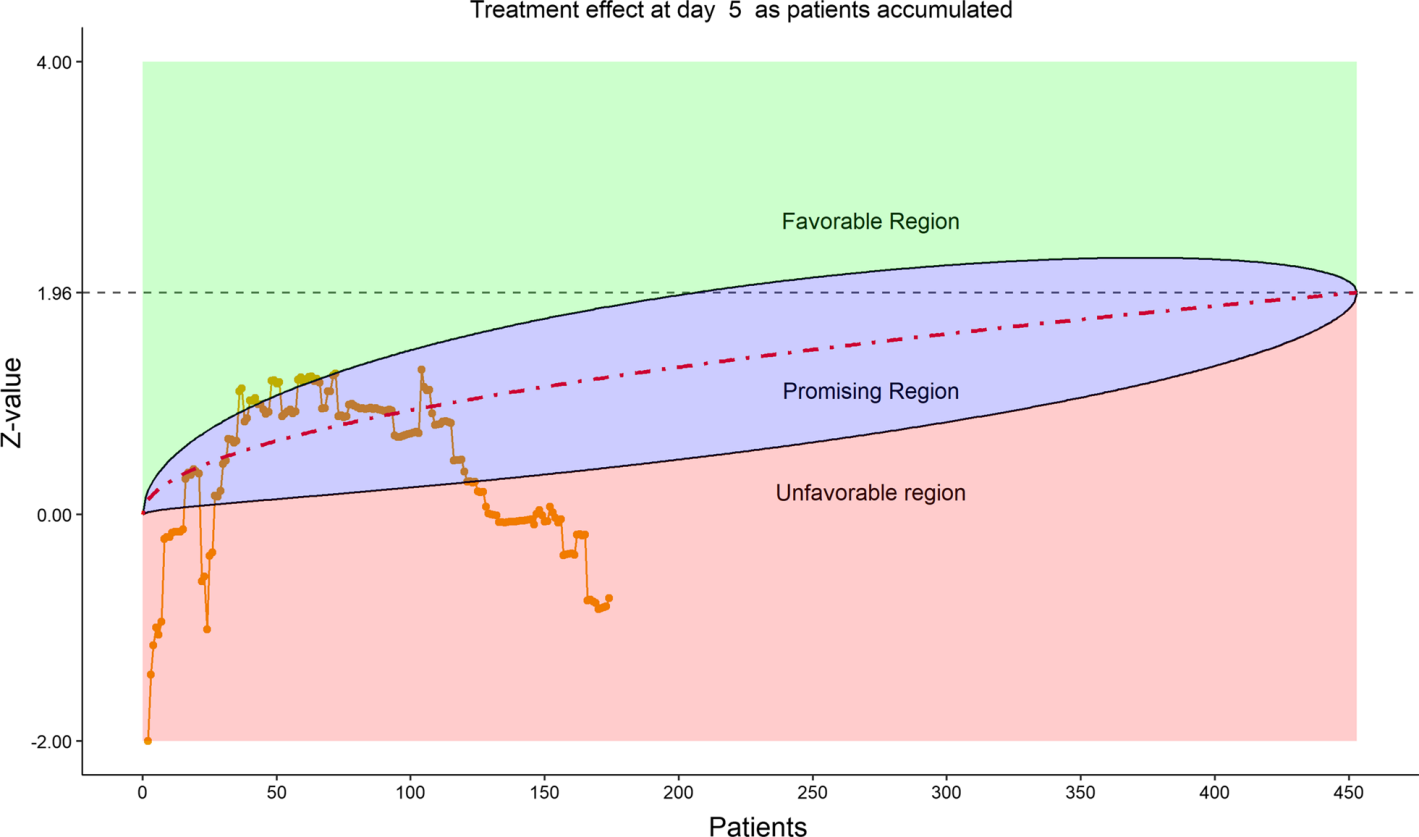
DSMB First Data Review: Dynamic Data Monitoring Z-Score from Stratified Wilcoxon–Mann–Whitney Rank-Sum Test Along Number of Patients Enrolled, On Day 5; The Upper Boundary of the “Promising Zone” Was Set for Conditional Probability (CP) = 90% and the Lower Boundary Was Set for CP = 5%. The Middle Dash Line Represented CP = 50%

**Figure 4:**
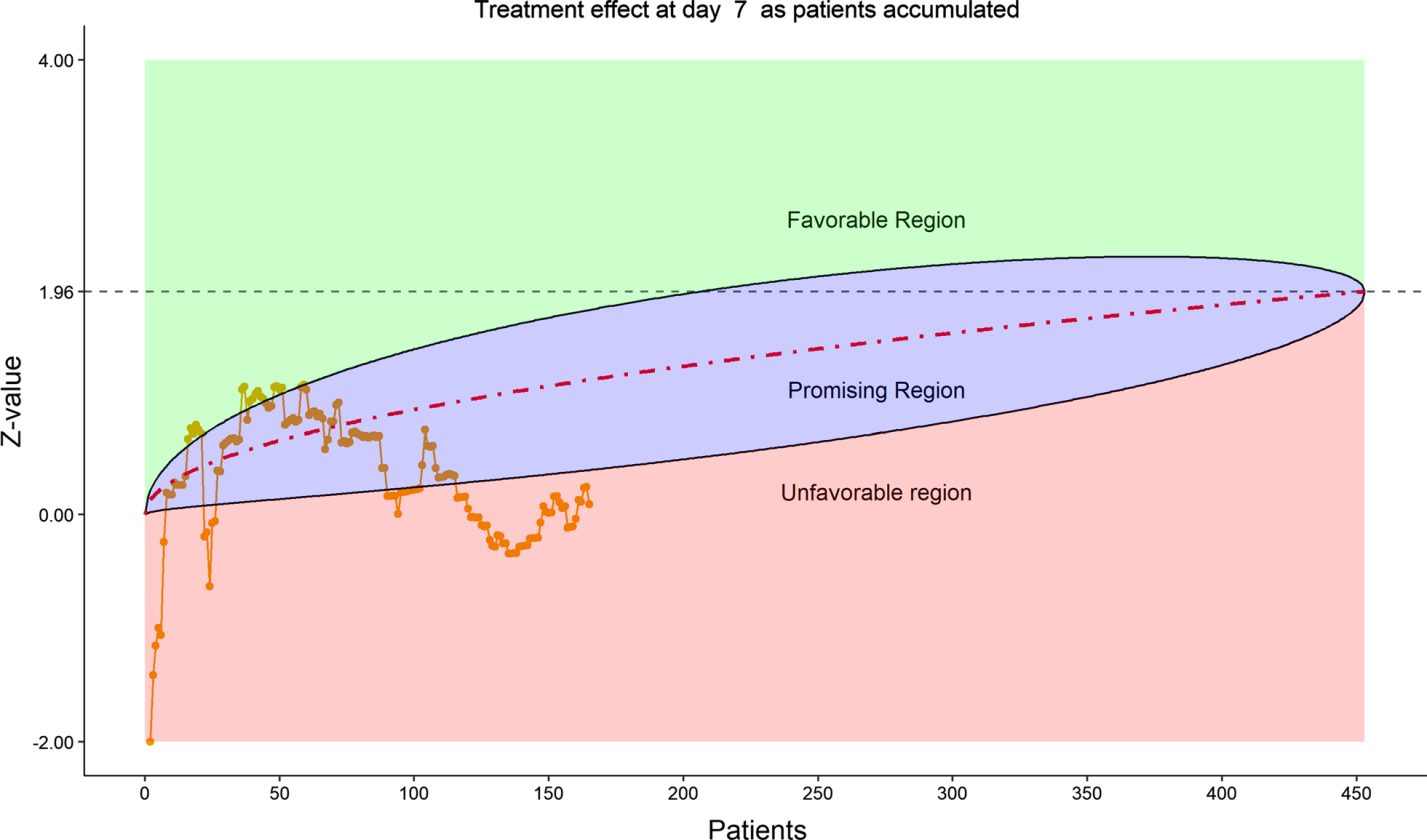
DSMB First Data Review: Dynamic Data Monitoring Z-Score from Stratified Wilcoxon–Mann–Whitney Rank-Sum Test Along Number of Patients Enrolled, on Day 7; The Upper Boundary of the “Promising Zone” Was Set for Conditional Probability (CP) = 90% and the Lower Boundary Was Set for CP = 5%. The Middle Dash Line Represented CP = 50%

**Figure 5:**
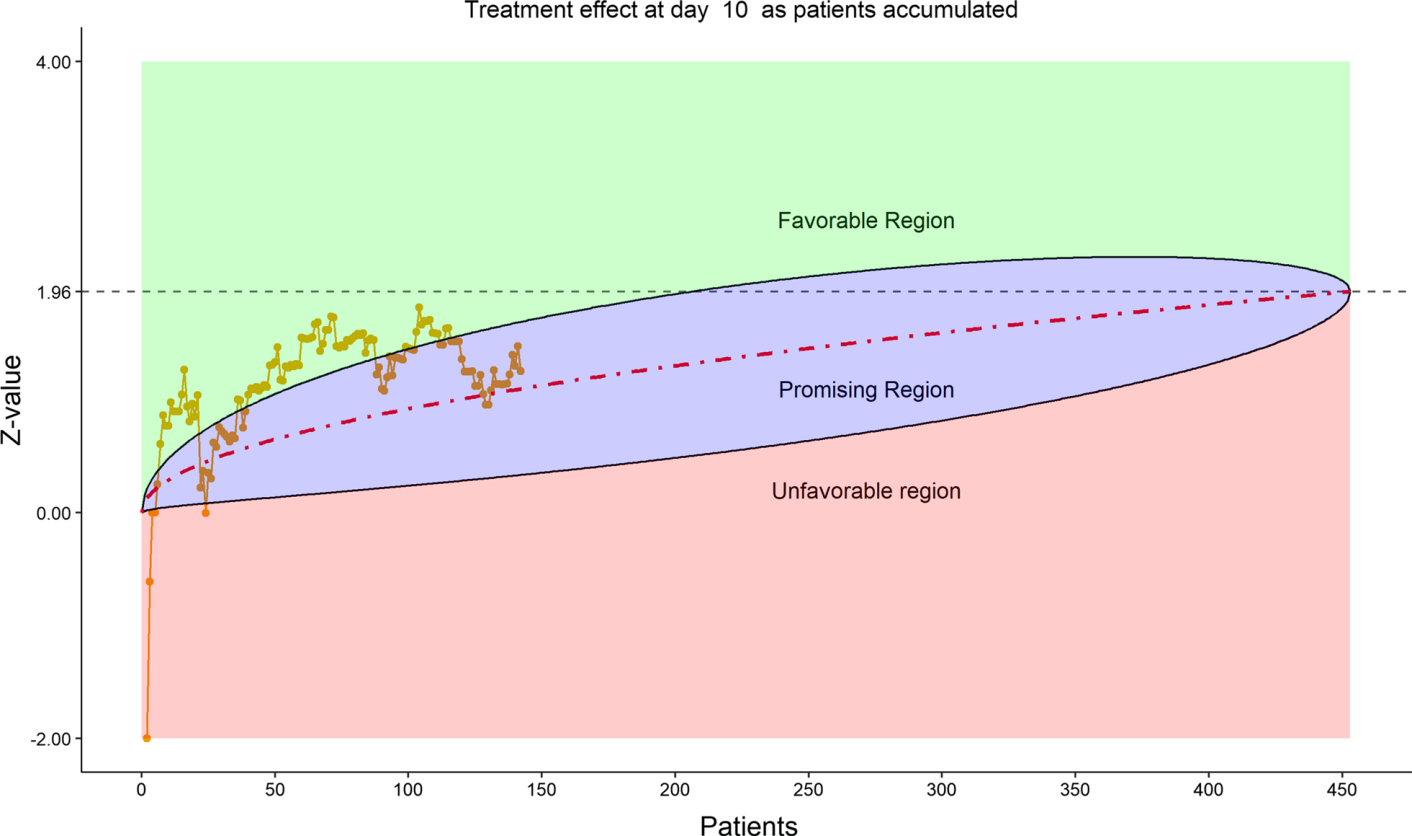
DSMB First Data Review: Dynamic Data Monitoring Z-Score from Stratified Wilcoxon–Mann–Whitney Rank-Sum Test Along Number of Patients Enrolled, on Day 10; The Upper Boundary of the “Promising Zone” Was Set for Conditional Probability (CP) = 90% and the Lower Boundary Was Set for CP = 5%. The Middle Dash line Represented CP = 50%

Figures 2, 3, 4, and 5 show the dynamic graphs on the movement of the stratified WMW rank-sum test (*Z*-value) as patient enrollments accumulated on each of the post-randomization Days 3, 5, 7, and 10, respectively. The upper boundary of the “promising zone” was set for conditional probability (CP) = 90% and the lower boundary was set for CP = 5%. The middle dash line represented CP = 50%. As shown, remdesivir did not have a quick effect on the clinical scale compared to the placebo (on Days 3, 5, 7), but was trending upward on Day 10. This fact provided a hopeful scenario for the DSMB to recommend continuing the trial and planning for the next DSMB meeting a week later. Note that, unlike Day 7, the data on the Days 3, 5, and 10 were unplanned but requested by and provided to the DSMB instantaneously with the efficient eDMC system. The supplementary data provided a useful “trend” information for the DSMB review. In addition, at this time, the COVID-19 epidemic in Wuhan was slowing down, and many other studies also started in the region in February, competing for the patient resources. Facing the decline of enrollment, DSMB recommended the investigators to consider enhancing their enrollment effort and to the trial sponsor to re-evaluate the planned time-line for study completion date (the original projection date was April 27, 2020).

DSMB also urged the investigators to verify the patient’s eligibility criteria, especially the baseline SPO2 and/or the PAO2/FIO2 levels for this trial of severe COVID-19.

Aside: On February 21, 2020, the US National Institute of Allergy and Infectious Diseases (NIAID) launched a multicenter, adaptive, randomized, blinded, controlled trial involving remdesivir for the treatment of COVID-19 in hospitalized adults [10], as the disease was spreading fast globally. The remdesivir regimen was the same as in the Chinese trials. DSMB noticed that the NIAID trial used a similar 8-category ordinal scale for the trial’s primary endpoint. However, the in-patient follow-up time was 15 days, shorter than the 28 days as in the Chinese trials. Sample size was *n* = 440, similar to the sample size *n* = 453 for the Chinese trial with severe cases.

### The Third DSMB Meeting (Second Data Review): February 29, 2020

Prior to the meeting, the investigator team communicated that the patient enrollment to the trial of the mild–moderate cases had also been adversely impacted by the decline of COVID-19 epidemic, which was fortunate for the disease control, but sorry news for the trial per se. The focus of DSMB review remained on the first trial with severe cases.

There were 228 patients randomized (152 in the remdesivir group and 76 in the placebo group). Only 13 new patients were enrolled since the last DSMB review 7 days ago. However, more patients had completed the 10 days treatment and reached 14 days post-treatment follow-up. With carrying-over the status of discharged and death, the 6-category clinical scale showed 201 patients with data on Day 10, 177 patients on Day 14, and 97 patients on Day 21. Still, most (191/228, or 83.8%) were with baseline category = 3.

For safety, total deaths = 24 (10.53% mortality), evenly distributed between the two treatment groups. No concerned AEs were found, but DSMB noticed that more patients in the remdesivir group discontinued treatment than the placebo group (13/149, 8.7% versus 3/75, 4.0%). However, 20/149 (13.4%) of the remdesivir-treated patients had serious AEs, less than the placebo group (15/75, or 20.0%).

**Figure 6:**
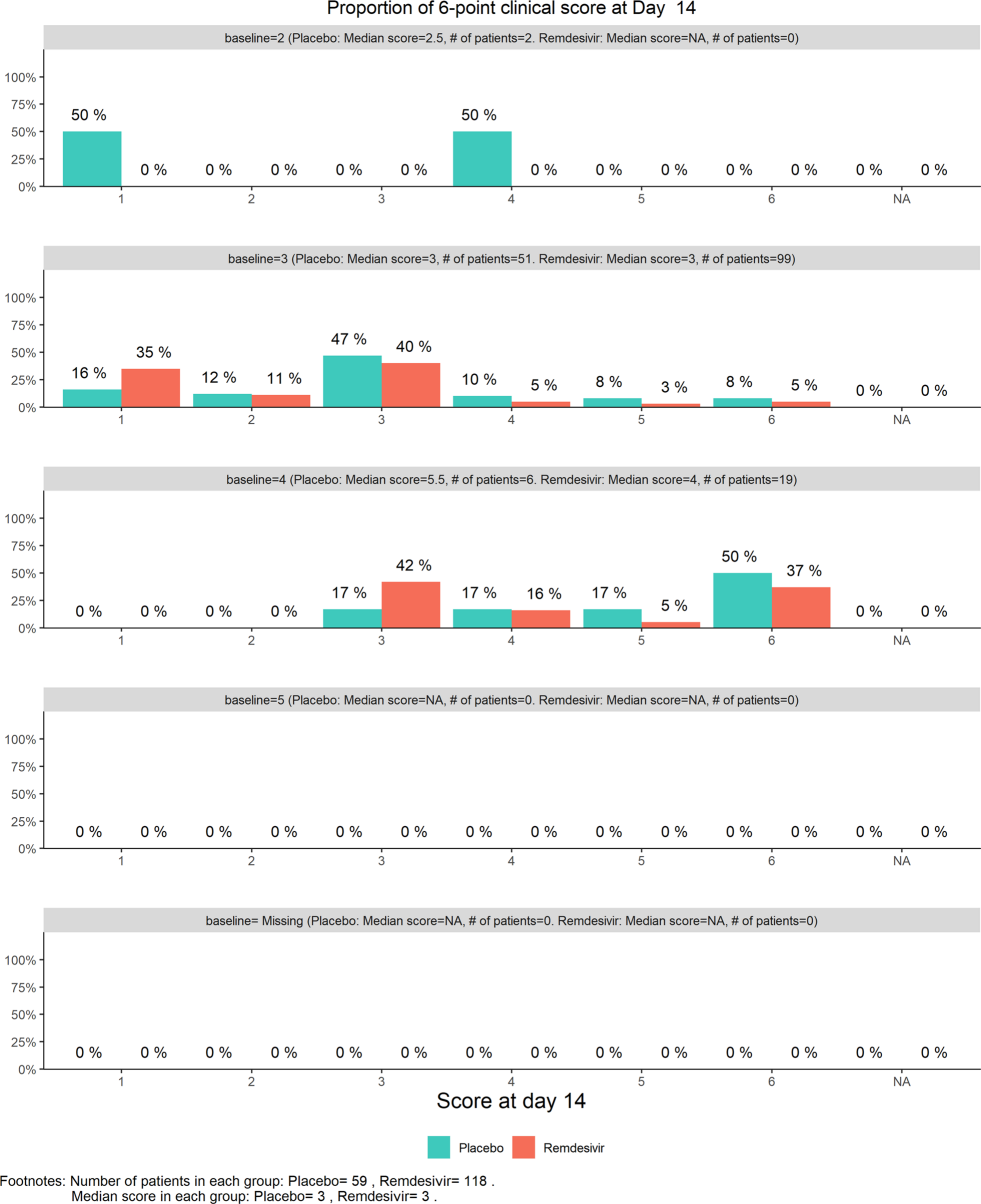
DSMB Second Data Review: Distribution of 6-Category Scale by Baseline Score at Day 14. Green Color is Placebo, Red Color is Remdesivir. Number of Patients in Each Group: Placebo 59, Remdesivir 118. Median Score in Each Group: Placebo 3, Remdesivir 3

**Figure 7:**
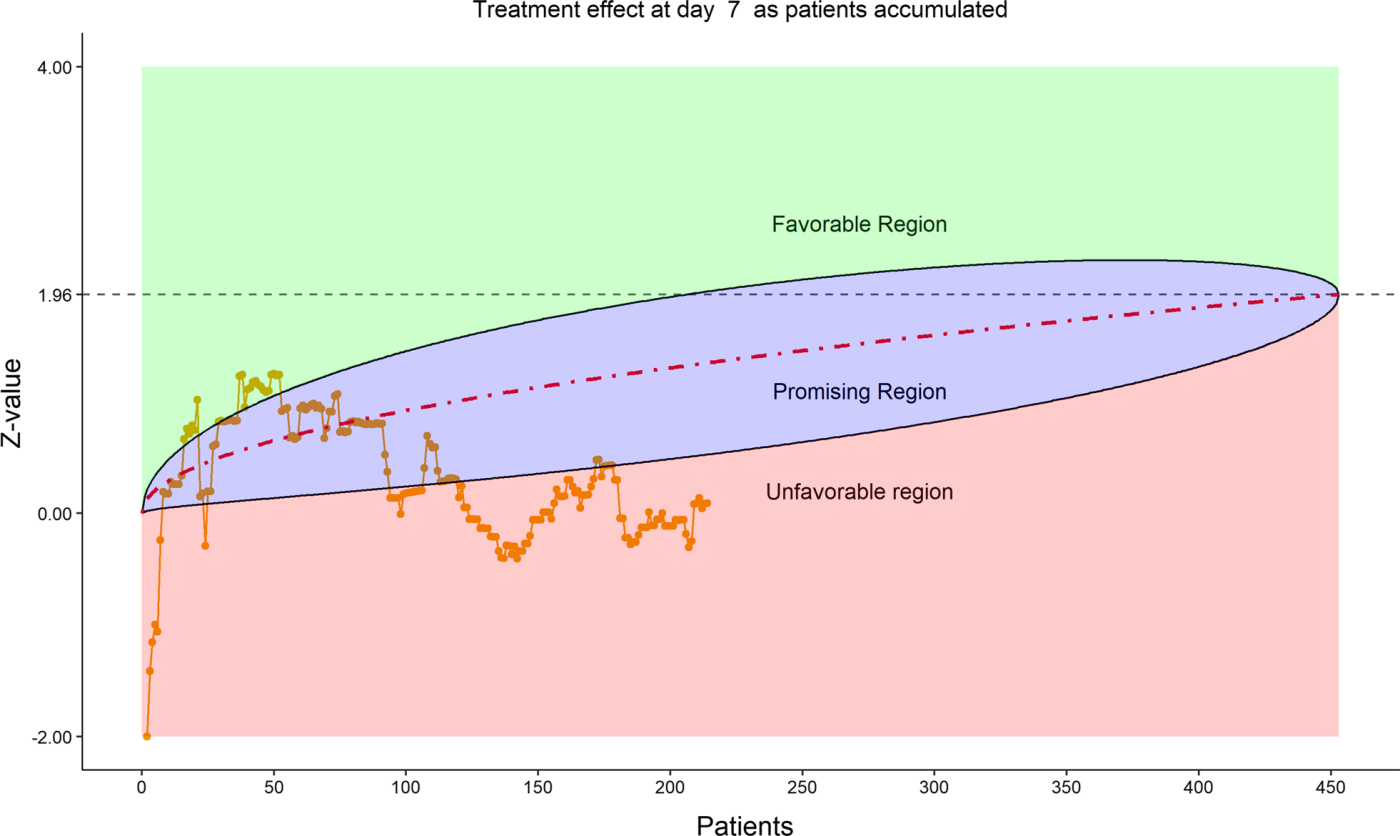
DSMB Second Data Review: Dynamic Data Monitoring Z-Score from Stratified Wilcoxon–Mann–Whitney Rank-Sum Test Along Number of Patients Enrolled, on Day 7; the Upper Boundary of the “Promising Zone” Was Set for Conditional Probability (CP) = 90% and the Lower Boundary Was Set for CP = 5%. The Middle Dash Line Represented CP = 50%

**Figure 8:**
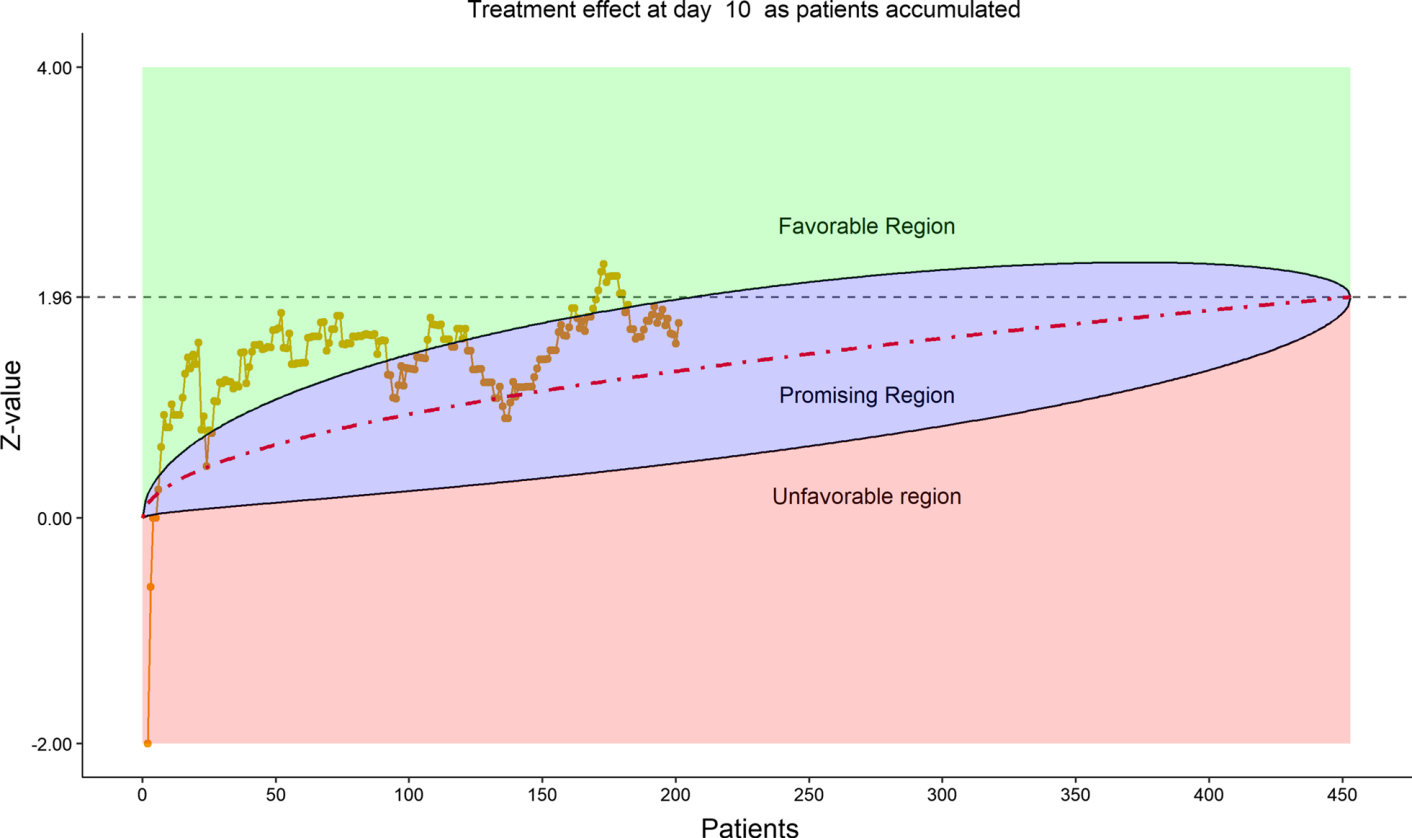
DSMB Second Data Review: Dynamic Data Monitoring Z-Score from Stratified Wilcoxon–Mann–Whitney Rank-Sum Test Along Number Of Patients Enrolled, on Day 10; The Upper Boundary of the “Promising Zone” Was Set for Conditional Probability (CP) = 90% and the Lower Boundary Was Set for CP = 5%. The Middle Dash Line Represented CP = 50%

**Figure 9:**
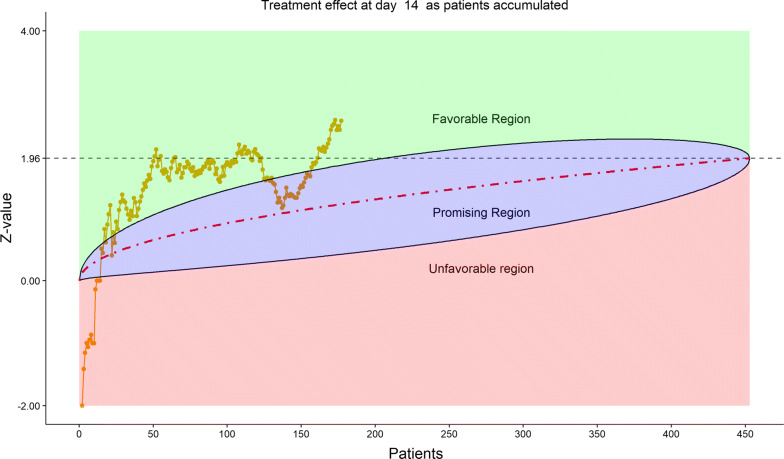
DSMB Second Data Review: Dynamic Data Monitoring Z-Score from Stratified Wilcoxon–Mann–Whitney Rank-Sum Test Along Number of Patients Enrolled, on Day 14; the Upper Boundary of the “Promising Zone” Was Set for Conditional Probability (CP) = 90% and the Lower Boundary Was Set for CP = 5%. The Middle Dash Line Represented CP = 50%

**Figure 10:**
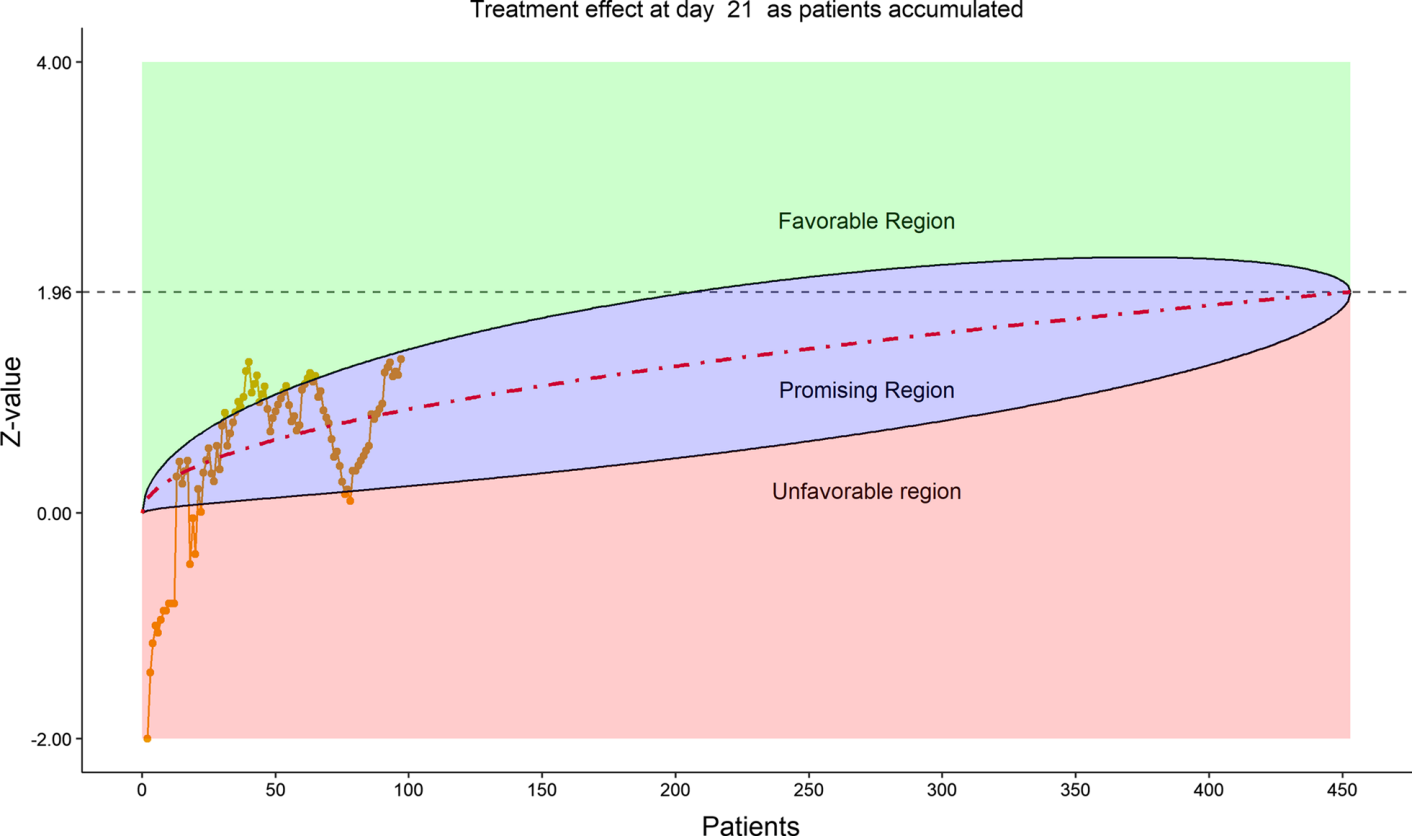
DSMB Second Data Review: Dynamic Data Monitoring Z-Score from Stratified Wilcoxon–Mann–Whitney Rank-Sum Test Along Number of Patients Enrolled, on Day 21; the Upper Boundary of the “Promising Zone” Was Set for Conditional Probability (CP) = 90% and the Lower Boundary Was Set for CP = 5%. The Middle Dash Line Represented CP = 50%

For efficacy, the total number of discharged from hospital was 55 (24.12%), slightly higher in the remdesivir group (40/152, or 26.32%) than in the placebo (15/76, or 19.74%). The 6-category clinical scale analysis added more enthusiasm for the DSMB as shown by the stratified distribution at Day 14 (bar chart, Fig. 6; other bar charts on earlier days were also examined by DSMB but omitted here), as well as by the dynamic “radar” graphs of the stratified WMW rank-sum tests at Days 7, 10, 14, and 21 (Figs. 7, 8, 9, 10, respectively; other graphs on earlier days were also examined by DSMB but omitted here). Based on the Z-value entering the favorable region on Day 14 (see Fig. 9), DSMB requested Kaplan–Meier plot (without p-value) be displayed for the TTCI endpoint at the next review meeting. Altogether, the tests seemed to be moving toward a favorable direction for remdesivir at this point in time, but the DSMB remained cautious on the yet to harvest data, especially since the Day 21 data were not adequate and lacked a trend in harmony with Day 14. The recommendation was to continue the trial, but DSMB urged the sponsor to re-evaluate the time-line for study completion date and the original planned interim analysis strategy on the primary endpoint, TTCI.

Aside: The protocol planned *n* = 453 severely ill patients to complete the 28 days study by April 28, 2020. With *n* = 228 randomized as of 2/28/2020, DSMB informed the sponsor that in the month of March, the investigator team must randomize 7 or 8 patients per day on average in order to complete the target enrollment at the end of March.

### The Fourth DSMB Meeting (Third Data Review): March 8, 2020

There were 235 patients randomized (157 in the remdesivir group and 78 in the placebo group). Only 7 new patients were enrolled from the last DSMB review 8 days ago. Baseline characteristics were comparable between the two groups. Among the 235, 217 (92.3%) had nasal catheter or mask, 27 (11.5%) had high flow oxygen, 15 (6.4%) had noninvasive ventilation, and 2 (0.9%) had invasive ventilation for their respiratory support before randomization. The median day of nasal catheter or mask was 3 days and high flow oxygen support was 2 days. Four patients withdrew their informed consent.

The baseline SPO2 level was also similar between the two treatment groups. The in-room temperature, not requiring supplemental oxygen SPO2 range was 75 to 94% (*n* = 75, out of 235 patients; median = 92%). With supplemental oxygen, the range of SPO2 was 37 to 100% (*n* = 158, out of 235 patients; median = 93%). One patient (in the placebo group) had hypercapnia respiratory failure at baseline, whose SPO2 was 88%.

Of the safety data set (*n* = 230), 26/153 (17%) patients in the remdesivir group had SAE, compared to 17/77 (22.1%) in the placebo group; few SAEs were judged drug-related by the investigators (2 in each group). However, more patients discontinued treatment due to AE in the remdesivir group than the placebo group (17/153 or 11.1% versus 3/77 or 3.9%), as also noted in the previous DSMB review. The mortality rate was 27/230 (11.74%), slightly increased from the last review and was comparable between the two groups.

For efficacy, although only 7 new patients enrolled since last review, more patients had completed the 10 days treatment and reached Day 14 and Day 21 post-treatment follow-ups. Carrying over the status of discharged and death, the 6-category clinical scale cumulated 220 patients with data on Day 10, 218 patients on Day 14, 197 patients on Day 21, and 143 patients on Day 28. Still, mostly (192/230, or 83.5%) were with baseline category = 3. For this reason, DSMB firmly believed that the analysis of “clinical improvement” (as for the TTCI endpoint) would need to be supplemented by an analysis of “clinical no-change or worsening.” The DSMB’s analysis of the distribution of the 6-category scale using the stratified WMW rank-sum test was actually on-target.

**Figure 11:**
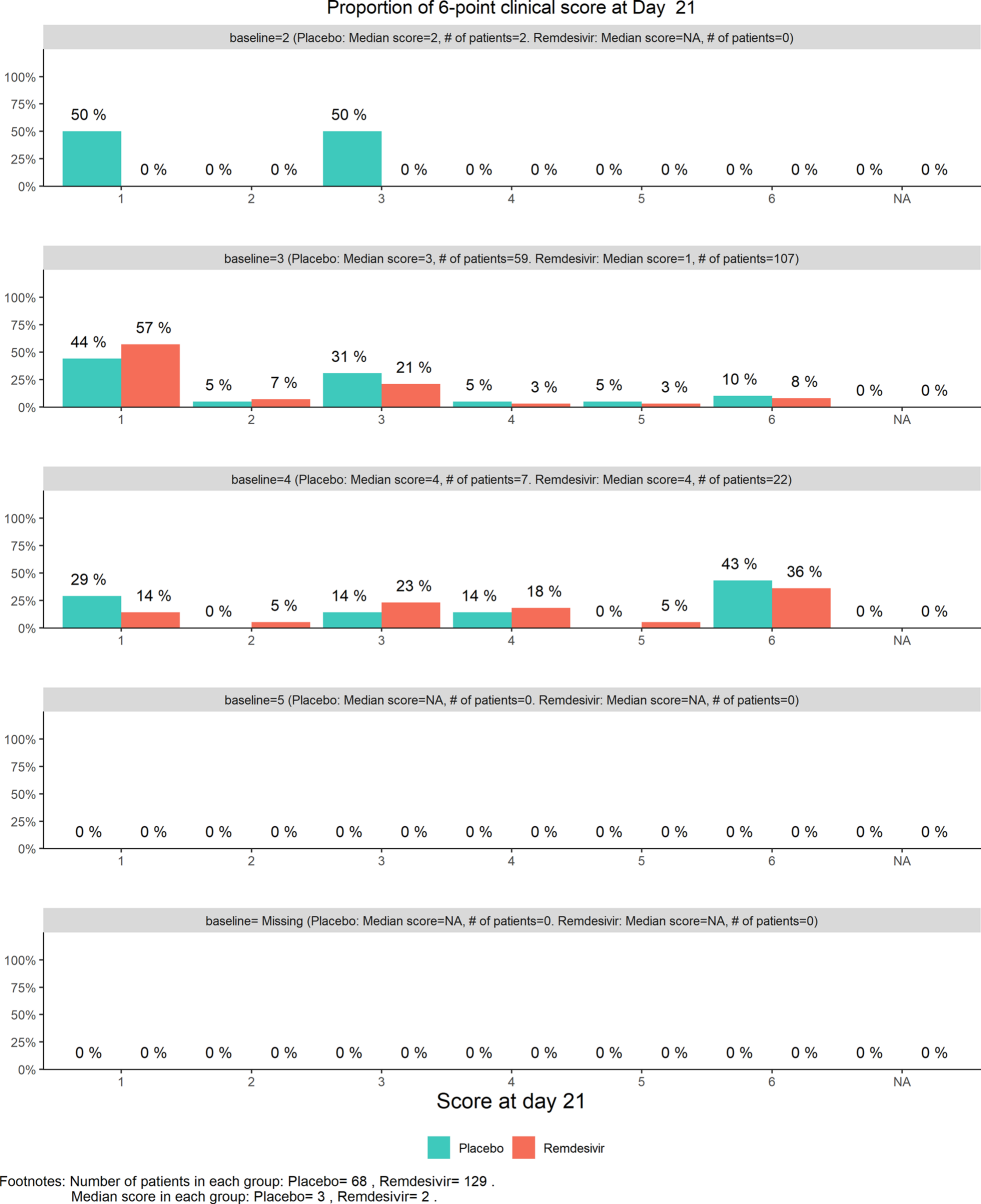
DSMB Third Data Review: Distribution of 6-Category Scale by Baseline Score at Day 21. Green Color Is Placebo, Red Color Is Remdesivir. Number of Patients in Each Group: Placebo 68, Remdesivir 129. Median Score in Each Group: Placebo 3, Remdesivir 2

**Figure 12:**
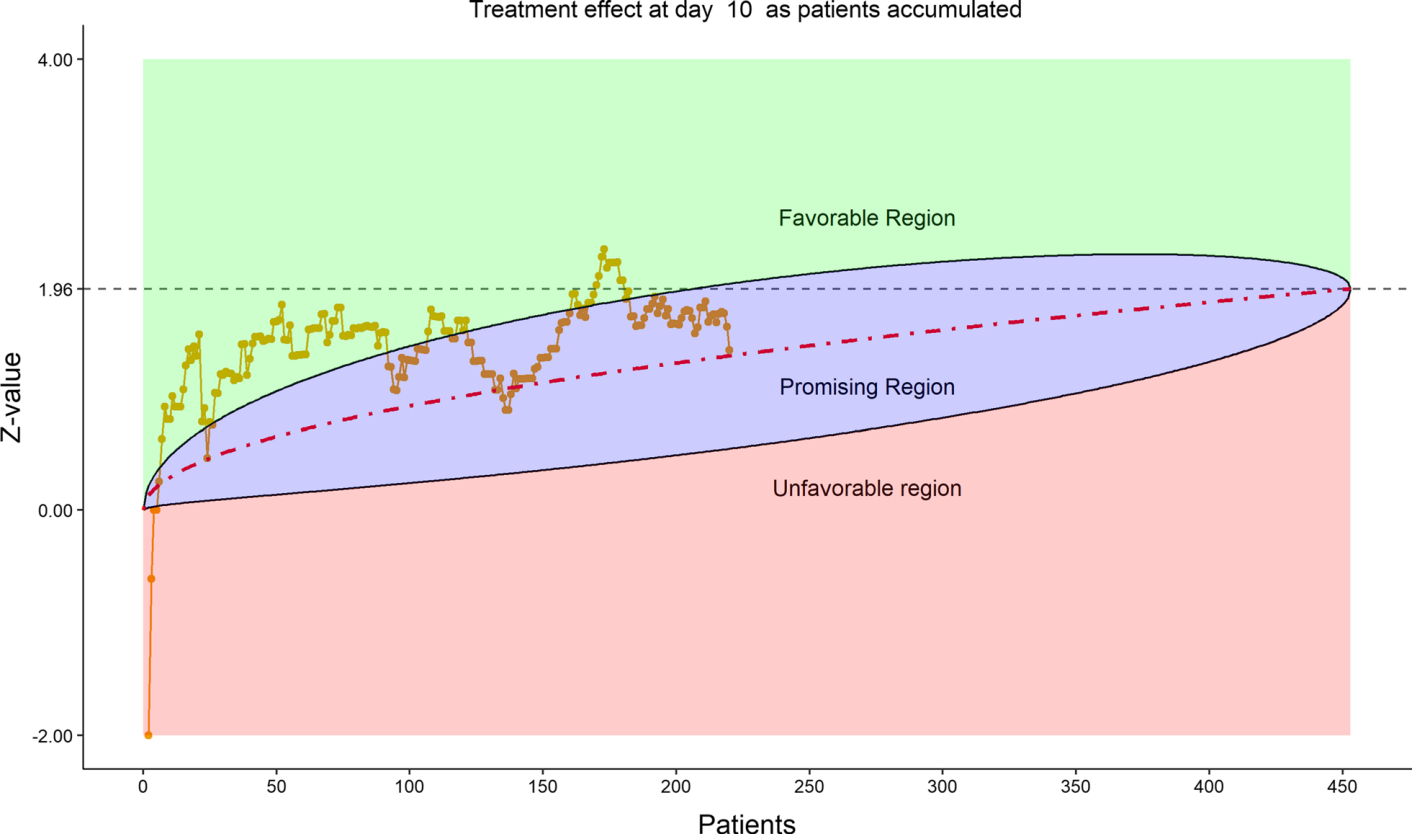
DSMB Third Data Review: Dynamic Data Monitoring Z-Score from Stratified Wilcoxon–Mann–Whitney Rank-Sum Test Along Number of Patients Enrolled, on Day 10. The Upper Boundary of the “Promising Zone” Was Set for Conditional Probability (CP) = 90% and the Lower Boundary Was Set for CP = 5%. The Middle Dash Line Represented CP = 50%

**Figure 13:**
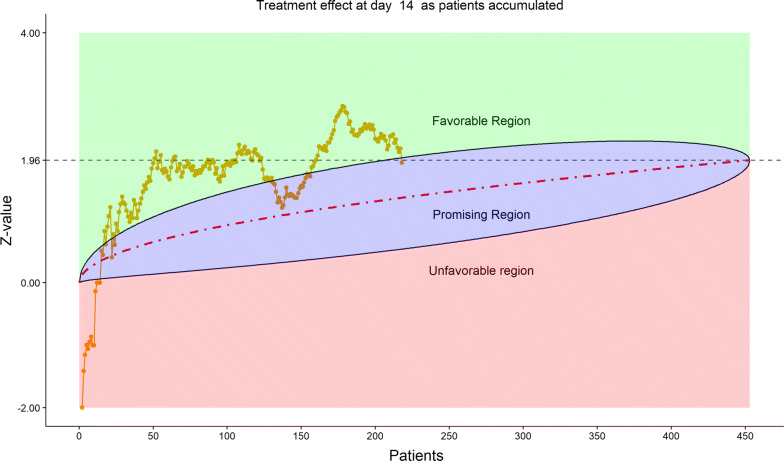
DSMB Third Data Review: Dynamic Data Monitoring Z-Score from Stratified Wilcoxon–Mann–Whitney Rank-Sum Test Along Number of Patients Enrolled, on Day 14. The Upper Boundary of the “Promising Zone” Was Set for Conditional Probability (CP) = 90% and the Lower Boundary Was Set for CP = 5%. The Middle Dash Line Represented CP = 50%

**Figure 14:**
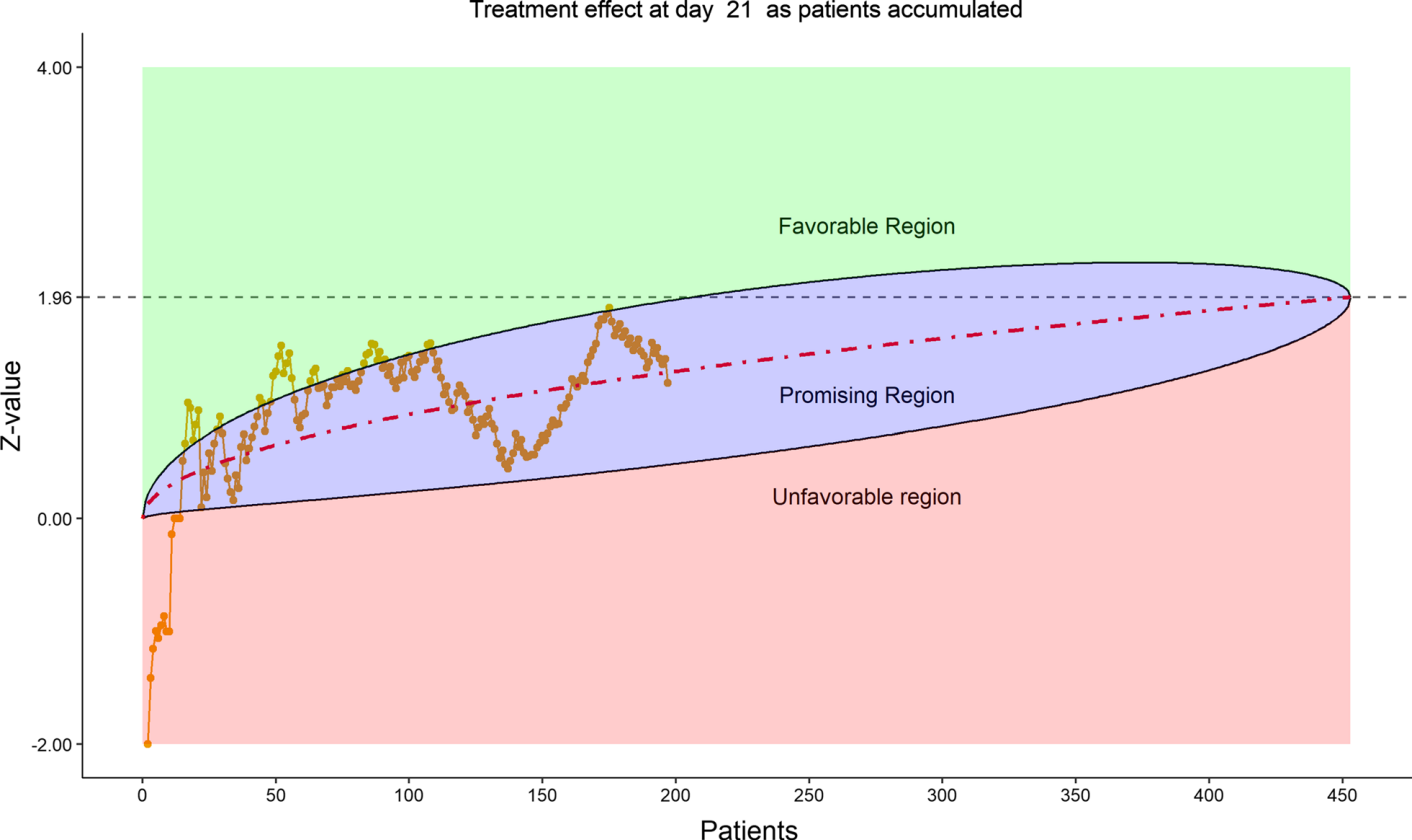
DSMB Third Data Review: Dynamic Data Monitoring Z-Score from Stratified Wilcoxon–Mann–Whitney Rank-Sum Test Along Number of Patients Enrolled, on Day 21. The Upper Boundary of the “Promising Zone” Was Set for Conditional Probability (CP) = 90% and the Lower Boundary Was Set for CP = 5%. The Middle Dash Line Represented CP = 50%

**Figure 15:**
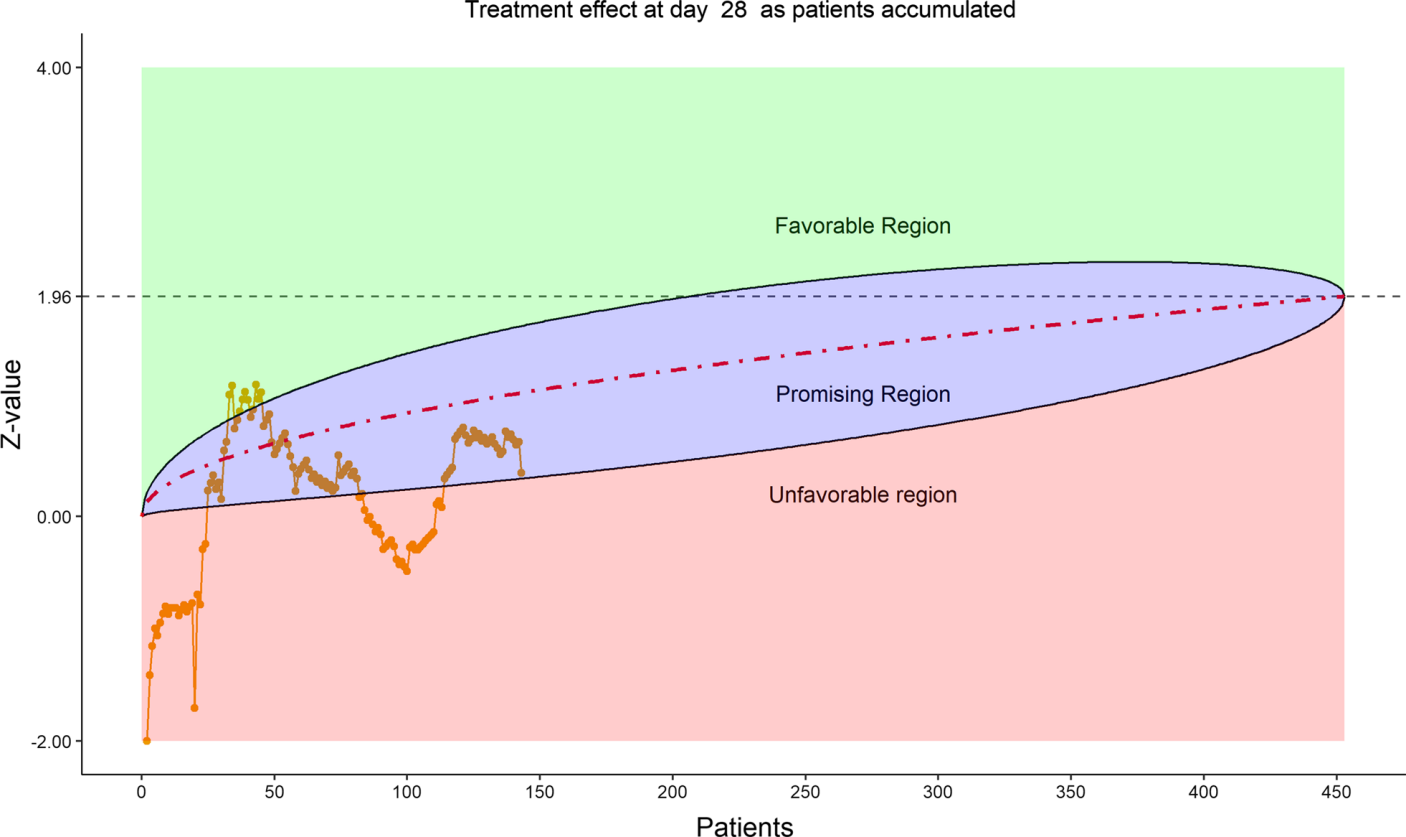
DSMB Third Data Review: Dynamic Data Monitoring Z-Score from Stratified Wilcoxon–Mann–Whitney Rank-Sum Test Along Number of Patients Enrolled, on Day 28. The Upper Boundary of the “Promising Zone” Was Set for Conditional Probability (CP) = 90% and the Lower Boundary Was Set for CP = 5%. The Middle Dash Line Represented CP = 50%

The total number of discharged from hospital was 92 (40.0%), lower in the remdesivir group (58/153, or 37.91%) than in the placebo group (34/77, or 44.16%). This reversal from the previous data review worried the DSMB. Less optimistic than before, DSMB examined the 6-category clinical scale data, which were also disappointing in the following sense, as shown by the stratified distribution at Day 21 (bar chart, Fig. 11; other bar charts on earlier days were also examined by DSMB but omitted here), as well as by the dynamic “radar” graphs of the stratified WMW rank-sum tests at Days 10, 14, 21, and 28 (Figs. 12, 13, 14, 15, respectively; other graphs on earlier days were also examined by DSMB but omitted here). Rather than maintaining in the favorable region on the “radar” screen, Day 14 rank-sum test *Z*-value seemed moving downward. Moreover, rather than strengthening the Day 14 result, Day 21 and Day 28 tests seemed moving away from favorable region for remdesivir. For the first time, the eDMC system, at the request of the DSMB from the last meeting, displayed the TTCI’s Kaplan–Meier plot (without p-value); the median TTCI was about 23 days versus 24 days for the remdesivir and placebo groups, respectively. The 1-day difference was much lower than the 6-day difference expected in the trial protocol.

Learning from the sponsor that, due to the continuing decline of COVID-19 cases in Wuhan, investigators would not be able to enroll new patients in order to reach the protocol-planned number, DSMB recommended sponsor to consider expanding the study to other cities in China or to other countries. Otherwise, sponsor needs to revise the protocol: (a) change the planned sample size for the declining enrollment reason; (b) cancel the scheduled interim analysis of TTCI; (c) continue the trial till all randomized patients finish their 28 days follow-up; and (d) prepare the final DSMB review meeting at the end of March for the trial with severe cases.

Aside: On March 3, 2020, Gilead Science launched two clinical trials of remdesivir [11, 12]. For the moderate cases [11], the primary endpoint was time to discharge in 5 to 10 days. For the severe cases [12], the primary endpoint was proportion of participants with normalization of fever and oxygen saturation through Day 14. On March 11, 2020, the World Health Organization declared the rapidly spreading COVID-19 outbreak a pandemic.

### The Fifth DSMB Meeting (Fourth And Final Data Review): March 29, 2020

DSMB received information from the sponsor that our last recommendation of revising the protocol of the first trial with severe cases was accepted. Hence, this would be the last DSMB review on the data of the severe cases. The second trial of mild–moderate patients was still too early for analyzing the efficacy data; only baseline and safety data were presented by the CRO. Our focus was still on the severe cases as follows:

There were 237 patients randomized (158 in the remdesivir group and 79 in the placebo group). Only 2 new patients were enrolled from the last DSMB review three weeks ago. Of the 237, 195 (82.3%) completed the study, 1 was still on-going, 35 (14.8%) died, and 6 (2.5%) withdrew for reasons other than death. The 1 on-going patient was to complete the Day 28 follow-up by 4/1/2020.

The intent-to-treat all-cause mortality rate was 24/158 (15.2%) in the remdesivir group and 11/79 (13.9%) in the placebo group, including 2 patients who died after randomization prior to receiving remdesivir and 3 patients died after Day 28 (1 in placebo and 2 in remdesivir group). These rates were lower than the corresponding rates  (19.2% vs 25.0%) reported in the trial of lopinavir–ritonavir vs SOC by the same investigator team [13].

On the 6-category ordinal scale at baseline, the distributions (%) were (0, 0, 81.6, 17.7, 0, 0.6) for the remdesivir group and (0, 3.8, 83.5, 11.4, 1.3, 0) for the placebo group, in Categories 1 (discharged or met discharge criteria) to 6 (death). As seen, majority (> 80%) were Category 3 patients, who were hospitalized, required supplemental oxygen (but not NIV/ HFNC).

The live discharge rate continued to be lower for the remdesivir group (107/157, 68.2%) compared to 57/77 (74.0%) in the placebo group. (Three  missing data were noted.)

Although only two new patients enrolled since last review, more patients had completed the 10 days treatment and reached Days 14, 21, and 28 post-treatment follow-ups. Carrying over the status of discharged and death, the 6-category clinical scale cumulated 228 patients with data on Day 10 (one patient was missing on Day 10), 229 on Day 14, 227 patients on Day 21, and 225 patients on Day 28. (The 6-category data excluded the 2 deaths occurred prior to receiving treatment and 3 deaths occurred after Day 28.)

**Figure 16:**
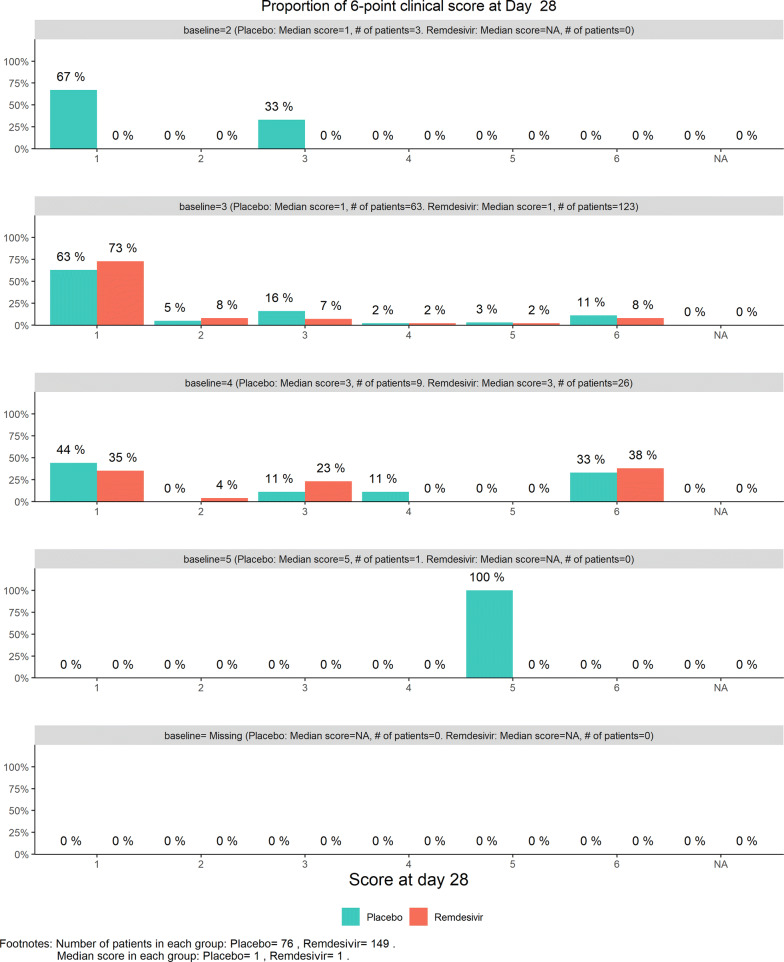
DSMB Fourth Data Review: Distribution of 6-Category Scale by Baseline Score at Day 28. Green Color Is Placebo, Red Color Is Remdesivir. Number of Patients in Each Group: Placebo 76, Remdesivir 149. Median Score in Each Group: Placebo 1, Remdesivir 1

**Figure 17:**
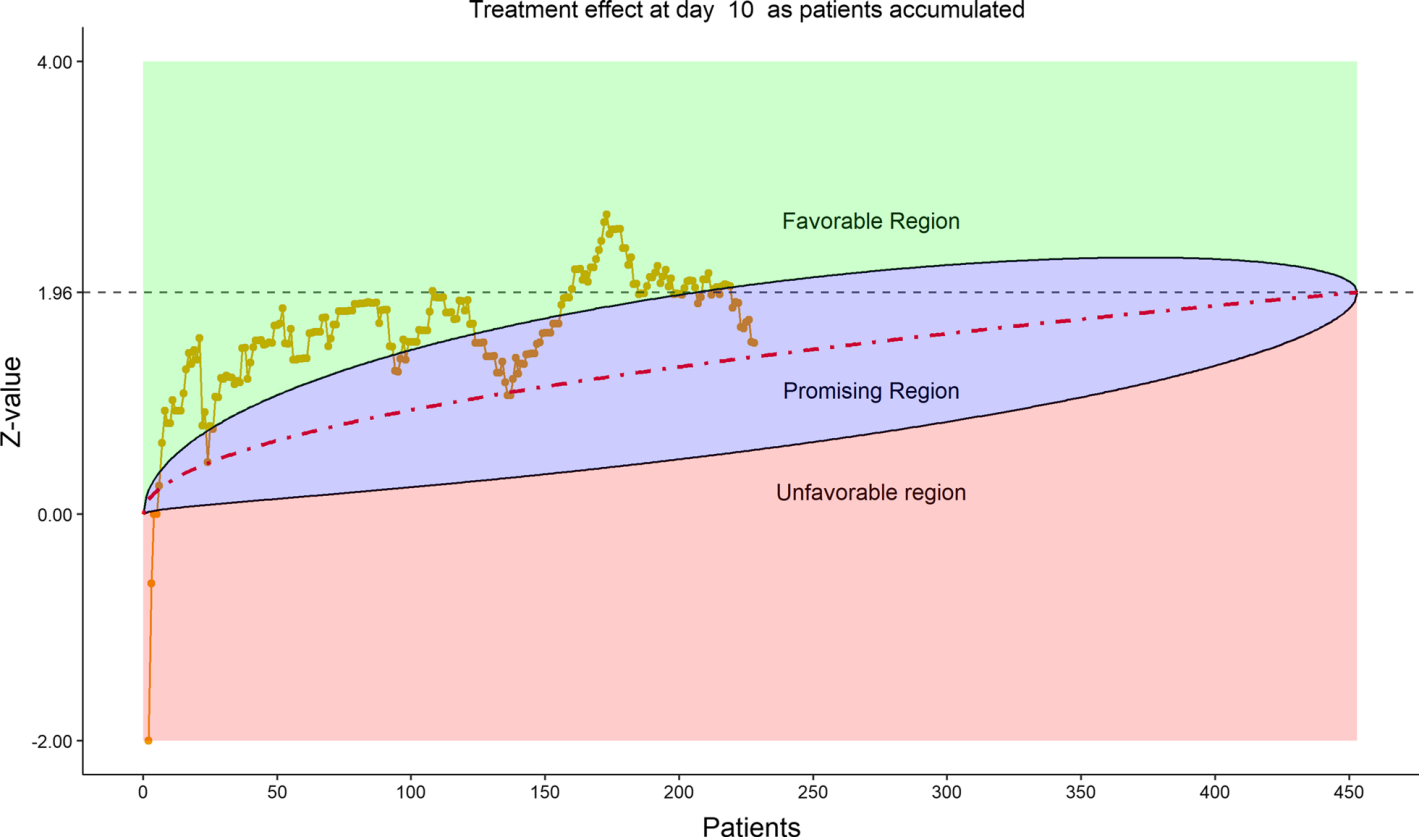
DSMB Fourth Data Review: Dynamic Data Monitoring Z-Score from Stratified Wilcoxon–Mann–Whitney Rank-Sum Test Along Number of Patients Enrolled, on Day 10. The Upper Boundary of the “Promising Zone” Was Set for Conditional Probability (CP) = 90% and the Lower Boundary Was Set for CP = 5%. The Middle Dash Line Represented CP = 50%

**Figure 18:**
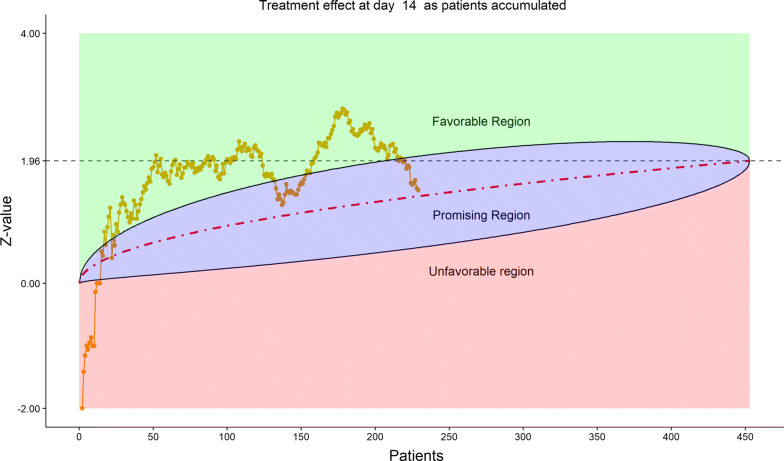
DSMB Fourth Data Review: Dynamic Data Monitoring Z-Score from Stratified Wilcoxon–Mann–Whitney Rank-Sum Test Along Number of Patients Enrolled, on Day 14. The Upper Boundary of the “Promising Zone” Was Set for Conditional Probability (CP) = 90% and the Lower Boundary Was Set for CP = 5%. The Middle Dash Line Represented CP = 50%

**Figure 19:**
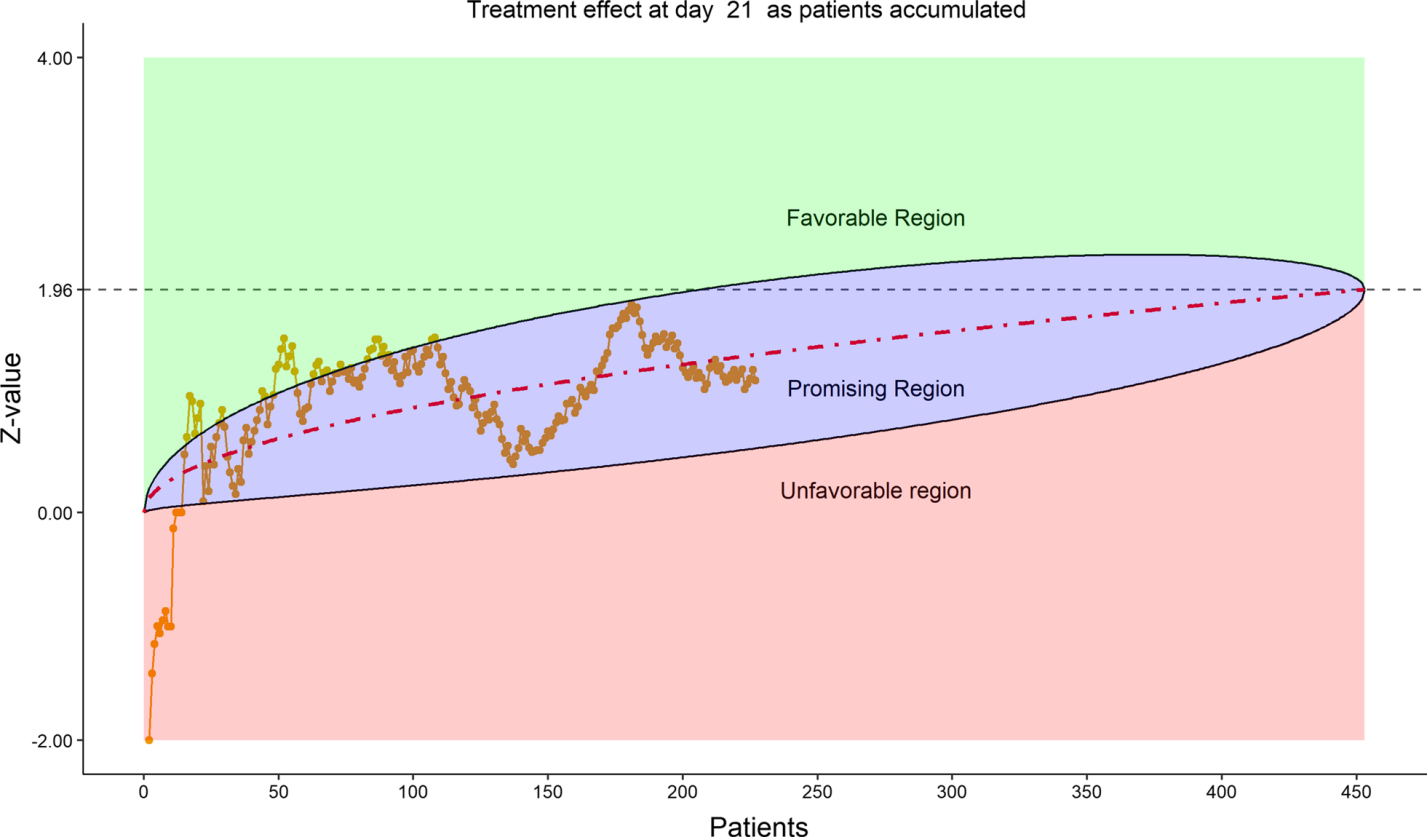
DSMB Fourth Data Review: Dynamic Data Monitoring Z-Score from Stratified Wilcoxon–Mann–Whitney Rank-Sum Test Along Number of Patients Enrolled, on Day 21. The Upper Boundary of the “Promising Zone” Was Set for Conditional Probability (CP) = 90% and the Lower Boundary Was Set for CP = 5%. The Middle Dash Line Represented CP = 50%

**Figure 20:**
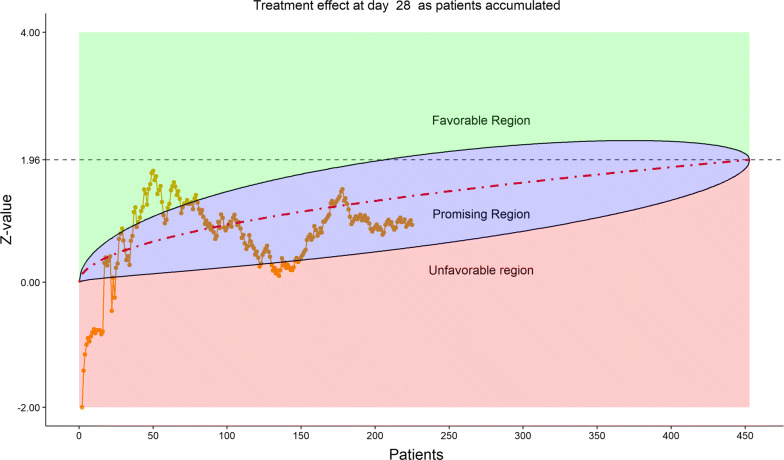
DSMB Fourth Data Review: Dynamic Data Monitoring Z-Score from Stratified Wilcoxon–Mann–Whitney Rank-Sum Test Along Number of Patients Enrolled, on Day 28. The Upper Boundary of the “Promising Zone” Was Set for Conditional Probability (CP) = 90% and the Lower Boundary Was Set for CP = 5%. The Middle Dash Line Represented CP = 50%

Figure 16 displays the stratified distribution of the 6-category scale on Day 28. (Other distribution bar charts on earlier days were also examined by the DSMB but omitted here). Figures 17, 18, 19, and 20 display the stratified WMW tests on the “radar” screen for Days 10, 14, 21, and 28. Other earlier days were also examined by the DSMB but not shown here. Similar trends were observed compared to the previous data review, but confirmed with more subjects. Z-values of the stratified WMW test on Days 10, 14, 21, and 28 were all in the “promising” region, with the conditional power just touching 50% from above on Days 10 and 14, then lowering below 50% (dash line) on Days 21 and 28. The conditional power was calculated to project the chance that the final Z-value would be ≥ 1.96 (unadjusted for multiplicity), if the trial were to continue to the original final sample size of 435 patients, given the current estimate of treatment effect.

The DSMB also reviewed the Kaplan–Meier plot (without *p*-value) of the TTCI endpoint. The median TTCI was 22 days for the remdesivir group versus 24 days for the placebo groups. The 2-day difference was disappointingly much smaller than the 6-day difference projected in the protocol.

In an ordinary situation, we would recommend continuing the trial, perhaps even with an increase of the sample size. However, it was unfortunate that the sponsor could not feasibly continue enrolling patients outside of China, where the pandemic had begun. Thus, the DSMB agreed to complete the study with the current cohort of 237 randomized patients. DSMB recommended to let the last on-going patient finish the Day 28 follow-up before unblinding. DSMB also urged the investigator team to conduct the final analysis as soon as possible after unblinding to share the trial results with the international community.

## Conclusion and Discussion

In conclusion, the international multi-disciplinary expert cooperation, and the use of Internet meeting and high efficiency data monitoring tools guaranteed the interest of study participants, satisfied the sponsor′s urgent need, and protected the integrity of the trials under the extraordinary circumstance. The formal interim analysis of TTCI was not triggered because there were not enough events and the study was terminated prematurely owing to the timely control of the COVID-19 epidemic in China. With the data from 237 patients, however, we believe valuable information can still be extracted for the study design as well as on the treatment effect of remdesivir on COVID-19 severe cases. The final medical report is to appear in Lancet [14] soon.^1^

For the final data reviewed on March 29, 2020, there were a numerically higher mortality rate and a lower hospital discharge rate against the remdesivir group. In addition, there were negative *Z*-values of the WMW rank-sum test on the 6-category scale observed in early Days 3, 5, and 7 on the “radar” screen. In contrast, there was 2-day shorter median TTCI in favor of the remdesivir group. One possible explanation for this inconsonance could be just random chance, since none of the numerical comparisons could pass the common statistical significance level, although no formal calculation was conducted. Another reason could be that the TTCI censored the deaths on Day 28, distorted the TTCI in spite of higher mortality. The third explanation could be that TTCI only looked at the improvement side and forgot the worsening side, while 83.5% of patients were in the middle of the 6-category scale, who could go either direction. Using TTCI as the primary endpoint was a weakness of the study design. A 28-day landmark logistic regression analysis with a binary endpoint of a properly defined “response” might be a better choice.

The 28 days follow-up duration was a strength of the trial design with severe cases. For the regimen of 10 days treatment, the data showed clearly that remdesivir did not have an immediate effect within the 7 days of treatment on the severe cases. The best effect on clinical improvement was shown on Day 14. This effect, however, did not seem to sustain to Days 21 and 28. The 10-day regimen might not be adequate for severe cases. If the trial were designed for only 14 days follow-up, this degrading of effect could not have been revealed. The trials [10, 12] conducted, respectively, by NIAID and Gilead Sciences were with 14-day duration.^2^

Lastly, our experience with using the DDM has demonstrated its efficiency and reliability in supporting DSMB’s instantaneous review of the secondary efficacy endpoint and key safety data during the emergent situation. DDM, when used properly by disciplined statisticians, has shown its capability of exploring the trial data flexibly and, in the meantime, protecting the trial’s scientific integrity.

## Footnotes

1 The medical report appeared on-line on April 29, 2020.

2 These two trials later revised their study durations.
